# Effect of a Developed Nursing Stretch Break Application on Work-Related Musculoskeletal Complications and Fatigue among Nurses: An Interventional Study

**DOI:** 10.1155/2022/7870177

**Published:** 2022-12-13

**Authors:** Elahe Hosseini, Roxana Sharifian, Azadeh Bashiri, Hadi Daneshmandi

**Affiliations:** ^1^Department of Health Information Management, School of Health Management and Information Sciences, Health Human Resources Research Center, Shiraz University of Medical Sciences, Shiraz, Iran; ^2^Department of Health Information Management, School of Health Management and Information Sciences, Health Human Resources Research Center, Clinical Education Research Center, Shiraz University of Medical Sciences, Shiraz, Iran; ^3^Research Center for Health Sciences, Institute of Health, Shiraz University of Medical Sciences, Shiraz, Iran

## Abstract

**Objective:**

The present study aimed to develop Nursing Stretch Break (NSB) application to relieve work-related musculoskeletal complications and fatigue among hospital nurses.

**Methods:**

This interventional study was conducted among 71 nurses (15 men and 56 women with a mean age of 33.32 ± 6.42) employed in Shiraz governmental hospitals, Southwest Iran, for four months. In this study, NSB was developed; it includes eight main menus for the user, including (1) Registration, (2) Stretches, (3) User panel, (4) Questions from the researcher, (5) About us, (6) Contact us, (7) Reports, and (8) Answers to questions. Data were collected pre and post-intervention via the Persian version of the Nordic Musculoskeletal Questionnaire (P-NMQ), the Persian version of the Multidimensional Assessment of Fatigue (P-MAF) Scale, the Persian version of the Numeric Rating Scale (P-NRS), and the Persian version of the Usefulness, Satisfaction, and Ease of use (P-USE) questionnaire.

**Results:**

NSB application usability testing showed that the mean scores of P-USE subscales were high. The prevalence of work-related musculoskeletal symptoms (WMSs) in the last 7 days in nurses' body parts, except the elbows and knees, was significantly lower after the intervention than before. In addition, the intensity of work-related musculoskeletal pain/discomfort in all body parts, except the knees, was significant relief after the intervention compared to before. The results demonstrated that the difference in the mean score of the total fatigue and its subscales, except “degree of interference with activities of daily living,” was significant after the intervention compared to before.

**Conclusions:**

NSB could be a low-cost and feasible ergonomic solution to improve the nurses' musculoskeletal health.

## 1. Introduction

Work-related musculoskeletal disorders (WMSDs) are a worldwide concern that develop in the soft tissue structures in the body, such as the muscles, tendons, joints, nerves, ligaments, cartilage, spinal discs, and blood vessels due to repeated or prolonged ergonomic exposures. Such exposures may include awkward or static postures, repetitive motions, forceful exertion, manual materials handling (MMH), contact stresses, vibration, temperature extremes, and lack of rest break, which may be exacerbated or modified by psychosocial and organizational issues and individual/personal characteristics [1]. WMSDs are considered a leading cause of worker impairment, absence from work, presenteeism, disability, compensation costs, and reduced productivity and performance at work [2].

A systematic review among African workers demonstrated that WMSD was a problem in many African countries, with the prevalence ranging from 15.0% to 93.6%. This study revealed that the highest reported symptoms were related to the “health professional cadres” [3]. The findings of a comprehensive study among the Iranian workforce and various job groups revealed that the prevalence of work-related musculoskeletal symptoms (WMSs) in the shoulders (56.1%) and knees (54.8%) was higher among “healthcare workers” as compared to those of other working groups [4].

Krishnan et al. reported that 97.3% of the nurses complained of having work-related pain during the last 12 months. The highest prevalence of WMSs was linked to the lower back (86.7%), ankles (86.7%), neck (86.0%), shoulders (85.0%), lower legs (84.7%), and upper back (84.3%). In addition, the participants complained of severe pain in the lower back (19.7%), right shoulder (29.7%), and left shoulder (30.3%). It is revealed that 73.1% of the nurses sustained WMSDs for the past 12 months [5]. Amin et al., in their study, reported that the most common body regions regarding WMSs were the neck, feet, upper back, and shoulders [6]. In another study, 97% of Chinese nurses reported experiencing at least one WMSD in the previous year. Low back pain was the most commonly reported WMSD (80.1%), followed by neck (78.6%) and shoulder pain (70.4%) [7].

Yao et al., in their study, demonstrated that the prevalence of WMSDs among study nurses was 84% in all body regions in the previous year, with the highest prevalence in the neck (68.2%), followed by the waist (67.6%) and shoulder (54.6%). Statistical analyses showed that physical exercise was associated with WMSDs in nurses. Therefore, the lack of exercise significantly increased the risk of WMSDs [8].

Nurses, as a vital working group in the health care industry, are prone to WMSs. They are encountered with various risk factors, including a high volume of work, awkward postures, highly dynamic repetitive activities, and patient handling for long hours. In these situations, a high prevalence of WMSs and a high level of fatigue are expected among hospital nurses. In a previous study, we found out that the prevalence of WMSs and the amount of fatigue among Iranian nurses were high. The highest prevalence of the reported WMSs in the last 12 months was linked to the ankles/feet (81.8%), lower back (80.2%), knees (63.2%), upper back (62.8%), and shoulders (62.4%). In addition, the mean ± standard deviation of total fatigue related to the last 7 days was 32.46 ± 5.59, indicating moderate to high level of fatigue [9]. Nowadays, mobile health applications are widely used for education, data collection, remote monitoring services, emergency medical services, and other health services [10]. Recent advances in smart portable devices such as mobile phones have shown the great capability of facilitating and decreasing the cost, particularly in academic environments. Also, performance is promising to use the app for teaching purposes [11]. Chhabra et al. in their study claimed that health applications were promising tools for improving outcomes in patients suffering from various chronic conditions. In this study, a smartphone app (called Snapcare) for self-management of chronic low back pain (LBP) facilitated the increase in physical activity and brought about clinically meaningful improvements in pain and disability in patients with chronic low back pain [12]. A study by Lee et al. revealed that the smartphone-based exercise program improved the pain intensity and perceived physical health of office workers with neck pain [13].

Therefore, to relieve work-related musculoskeletal complications and fatigue, we decided to design an application, so that hospital nurses can use it easily in the workplace.

## 2. Materials and Methods

### 2.1. Study Design, Setting, and Population

This study is “Phase II” from a comprehensive study among Iranian nurses. In Phase I, 500 Iranian nurses, employed in Shiraz governmental hospitals, Southwest Iran, were examined via the Persian version of the Nordic Musculoskeletal Questionnaire (P-NMQ) to determine the prevalence of WMSs and the Persian version of the Multidimensional Assessment of Fatigue (P-MAF) Scale to determine the amount of fatigue. This phase (Phase I) revealed that the prevalence of WMSs and the amount of fatigue among the study population was high [9].

Therefore, in Phase II, we decided to design and use an application to relieve work-related musculoskeletal complications and fatigue among Iranian hospital nurses. Phase II was an interventional study carried out on 71 hospital nurses. The subjects were selected from 500 participants in “Phase I” via simple random sampling. The sample size was calculated by Cochran's equation (equation (1), *n* = 74). Three nurses were excluded from the study for various reasons, including acute musculoskeletal injury during the study (one person) and changing the job (two people). Finally, 71 individuals were included in the study (response rate = 96%).

(1) $$\begin{array}{c} n=\frac{Nz^{2}pq}{Nd^{2}+z^{2}pq}n=\frac{500\times 1.96^{2}\times 0.94\times 0.06}{\left(500\times \left(0.05^{2}\right)+{\left(1.96\right.}^{2}\times 0.94\times 0.06\right)}≅74, \end{array}$$

where *n*: sample size, *N*: statistical population size = 500, *z*: 1.96, *p*=0.94, *q* = 0.06, and *d* = 0.05.

The inclusion criteria of the study were being willing to participate in the study, having at least one year of work experience, having no history of acute musculoskeletal disorders in different body regions, having no surgical experience in the musculoskeletal system of the body, not having a second job, and having and being able to use a mobile phone.

Exclusion criteria were unwillingness to continue cooperation during the study, occurrence of acute musculoskeletal injury during the study, performance of professional exercises and use of special diets while studying, pregnancy of the female subjects during the study, and leaving or changing the job. These criteria were determined by asking single questions.

The study was approved by the Ethics Committee of Shiraz University of Medical Sciences (approval ID: IR.SUMS.REC.1398.219). In addition, this study was conducted according to the Helsinki Declaration and its later amendments [14]. All nurses voluntarily participated in this study after signing a written informed consent form.

### 2.2. Data Gathering Tools

In the current study, data were gathered using self-reporting as follows.

#### 2.2.1. Demographic/Occupational Questionnaire

This questionnaire included age, height, weight, job tenure, working h/day, gender, marital status, number of children, education level, smoking, and shift work.

#### 2.2.2. Persian Version of the Nordic Musculoskeletal Questionnaire (P-NMQ)

The NMQ initially developed by Kuorinka et al. provides reports of discomfort in body regions. It includes the neck, upper and lower back, and upper and lower limbs and determines if symptoms were present during the last 12 months and the last 7 days [15]. The psychometric properties of the P-NMQ were examined by Choobineh et al. [16].

#### 2.2.3. Persian Version of the Numeric Rating Scale (P-NRS)

NRS is a unidimensional measure of pain/discomfort intensity [1]. The psychometric properties of the P-NRS were examined by Nasiriani et al. [17].

#### 2.2.4. Persian Version of the Multidimensional Assessment of Fatigue (P-MAF) Scale

The MAF scale was developed by Belza et al. It contains 16 items that assess various aspects of fatigue. This tool is a self-administered questionnaire on four dimensions of fatigue, including “degree and severity,” “amount of distress it causes,” “its timing,” and “the degree to which fatigue interferes with daily living activities.” For rating items, a numerical rating scale (1–10) is used for items 1–14, and a categorical scale (1–4) is used for timing items 15 and 16. Finally, a Global Fatigue Index (GFI) was calculated. For GFI, the scores range from 1 to 50 (1 = no fatigue to 50 = severe fatigue) [18]. The participants were asked to reflect upon their fatigue experiences within the last 7 days. The psychometric properties of the P-MAF were examined by Choobineh et al. [19].

#### 2.2.5. Persian Version of the Usefulness, Satisfaction, and Ease of Use (P-USE) Questionnaire

The USE questionnaire initially developed by Lund measures the subjective usability of a product or service. The USE contains 30 items on a 7-point Likert scale (1 = strongly disagree to 7 = strongly agree) with a “N/A” option. The items belong to four dimensions: usefulness, ease of use, ease of learning, and satisfaction [20]. The psychometric properties of the P-USE were examined by Pishdad et al. [21].

### 2.3. The Stages of the Study

The current study was conducted in two stages, as follows.

#### 2.3.1. Stage I: Designing an Application to Present Proper Stretches

In this stage, we developed and reviewed the Nursing Stretch Break (NSB) application. The research team included medical informatics and health information management specialists, software engineers, physiotherapists, occupational therapists, ergonomics specialists, and sport sciences specialists. To this aim, first, after the search in scientific databases, including Science Direct, PubMed/Medline, Scopus, ProQuest, Google Scholar, Clinical Key, Cochrane, IranDoc (Iranian database), MagIran (Iranian database), and also textbooks in the field of physiotherapy, occupational therapy, ergonomics, and sport sciences, related exercise stretches were extracted by the research team. Then, an expert panel was held to discuss and determine the stretch type. Finally, 24 exercise stretches related to different body regions, including the upper limbs, trunk, and lower limbs, were found to be proper for hospital nurses. The expert panel also discussed application modules and appearance features such as icon size and color.

It is worth mentioning that the NSB stretches were planned based on the FITT principle (Frequency, Intensity, Time, and Type). In addition, it was also adjusted based on SMART (Specific, Measurable, Achievable, Relevant, and Time-bound) goals. The panel of experts consisted of an ergonomist, two physiotherapists, an occupational therapist, three experts in health information technology, and two nurses.

In the next step, a prototype of the NSB was constructed. It includes eight main menus for the user, including (1) Registration, (2) Stretches, (3) User panel, (4) Questions from the researcher, (5) About us, (6) Contact us, (7) Reports, and (8) Answers to questions. The expert panel reviewed the constructed prototype (as mentioned above), discussed pitfalls, and presented solutions. Then, the defects of the NSB were fixed, and the final version was developed. After designing the NSB application, we examined its usability from the experts' point of view via the P-USE questionnaire.

The prototype of the NSB was beta-tested two times among 10 nurses with smartphones (5 smartphones with the android operating system and 5 smartphones with IOS operating system). For the first time, the application was successfully installed for 8 out of the 10 enrolled nurses (80%); it was not successfully installed for 2/10 nurses (20%) due to incompatibility of their android version of the smartphone. In the second time, the smartphone operating system version was updated. In this step, the NSB was successfully installed for 10 nurses, and we asked them to test the app during the next two weeks.

#### 2.3.2. Stage II: Using NSB to Relieve Work-Related Musculoskeletal Complications and Fatigue among Nurses

Stage II of the study consisted of three steps, as follows.

*(1) Step I: Before Intervention*. In this step, the participants completed the questionnaires mentioned above (demographic/occupational questionnaire, P-NMQ to examine WMSs, P-NRS to measure musculoskeletal pain/discomfort, and P-MAF scale to assess various aspects of fatigue) in their workplace. Each subject filled out the questionnaire in his/her workplace.

Demographic/occupational questionnaire, P-NMQ, and P-MAF scales were filled out during the work shift. For assessing the musculoskeletal pain/discomfort intensity, the participants were required to rate P-NRS on Saturday, Monday, and Wednesday at the beginning and end of the work shift. Then, the difference between the P-NRS scores at the beginning and end of the shift during the three working days was calculated. The mean of differences was considered as work-related musculoskeletal pain/discomfort.

*(2) Step II: Intervention*. In the intervention step, the participants installed the NSB application on their smartphones. The NSB provided stretches to the study nurses every hour. They performed various stretches for 5 to 10 minutes. Individuals were asked to hold the stretches for 30 seconds at a moderate intensity [22]. The stretches offered to the participants every hour were a combination of stretches suitable for the neck and shoulders, upper limbs, trunk, and lower limbs. The duration of the intervention (using the application) was four consecutive months [23].

To ensure that nurses performed the stretches, one of the researchers regularly monitored them through NSB reporting, presence at the hospital, phone calls, and virtual networks.

*(3) Step III: After the Intervention*. After the intervention, the participants completed the questionnaires as mentioned in Step I at their workplace. Besides, the usability of the NSB from the nurses' point of view was examined using the P-USE questionnaire.

All evaluations were done in the same work shifts before and after the intervention to prevent the impact of “work shift” (as a confounding variable) on the study findings.

**Figure 1:**
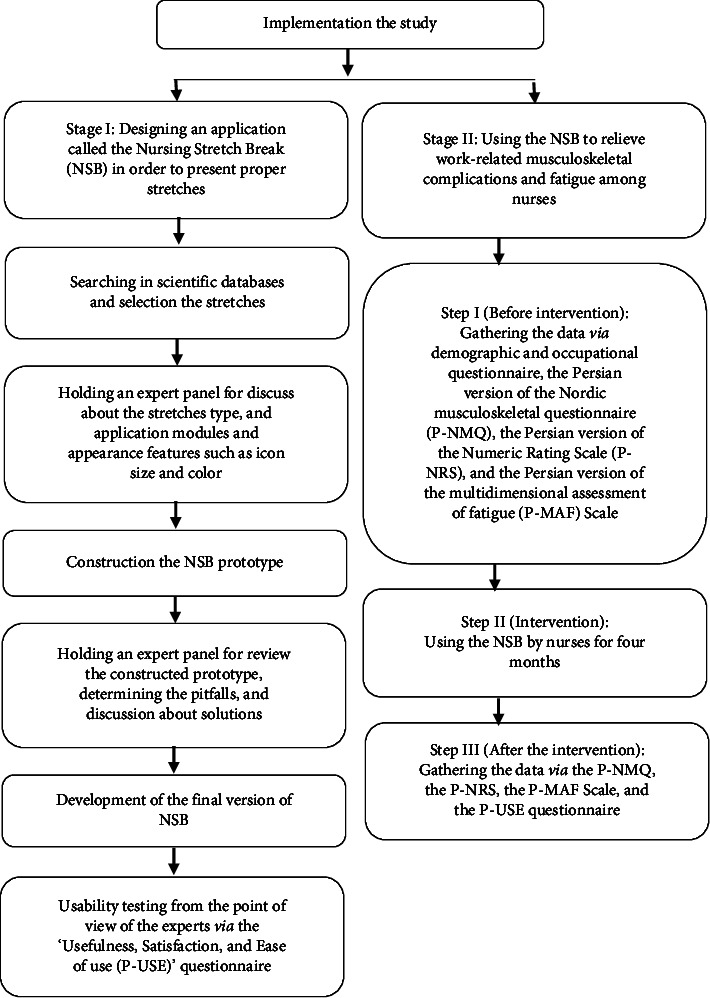
Flowchart of the study.

Figure 1 displays the flowchart of the study.

### 2.4. Statistical Analysis

Data were analyzed through the IBM SPSS version 21, using descriptive statistics, McNemar's test, paired sample *t*-test, and Wilcoxon signed-rank test. The Kolmogorov–Smirnov and Shapiro–Wilk tests were used to test the data normality. A *p* value <0.05 was considered to be statistically significant.

## 3. Results

The findings of the stages of the study were presented as follows.

### 3.1. Stage I: Designing an Application to Present Proper Stretches

Table 1 shows the usability testing of the NSB that was examined from the experts' point of view using the P-USE questionnaire.

**Figure 2:**
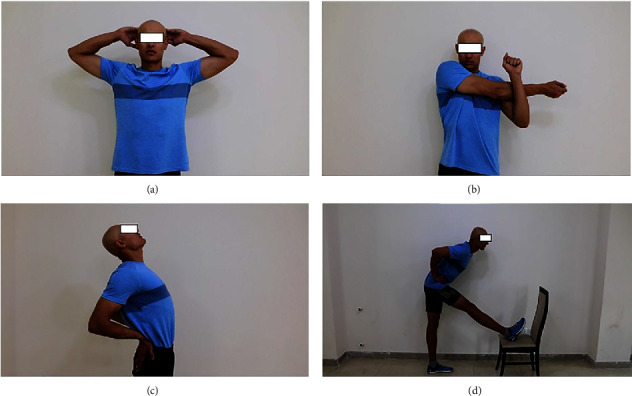
Some stretches included in the NSB: (a) above the head chest stretch, (b) cross-chest stretch, (c) mid-back stretch, and (d) standing hamstring stretch [22].

Figure 2 shows some stretches included in the NSB.

### 3.2. Stage II: Using the NSB to Relieve Work-Related Musculoskeletal Complications and Fatigue among Nurses

Table 2 shows the personal/occupational details of the nurses who participated in the study. As shown in the table, the means ± standard deviations of the subjects' age and job tenure were 33.32 ± 6.42 years and 9.70 ± 6.70 years, respectively.

Table 3 demonstrates the comparison of the study groups regarding the prevalence of WMSs in the last 7 days in different body regions before and after the intervention. As shown, the prevalence of WMSs in all body regions, except the elbows and knees, was significant before and after the intervention.

Table 4 compares work-related musculoskeletal pain/discomfort intensity in different body regions before and after the intervention. As shown, the intensity of work-related musculoskeletal pain/discomfort in all body regions, except the knees, was significant before and after the intervention.

Table 5 shows the comparison of the study groups' as to the total fatigue and its subscales before and after the intervention. As shown, the mean scores of the total fatigue and its subscales, except “degree of interference with activities of daily living,” were significant before and after the intervention.

Table 6 shows the usability testing of the NSB that was examined from the nurses' point of view using the P-USE questionnaire.

**Figure 3:**
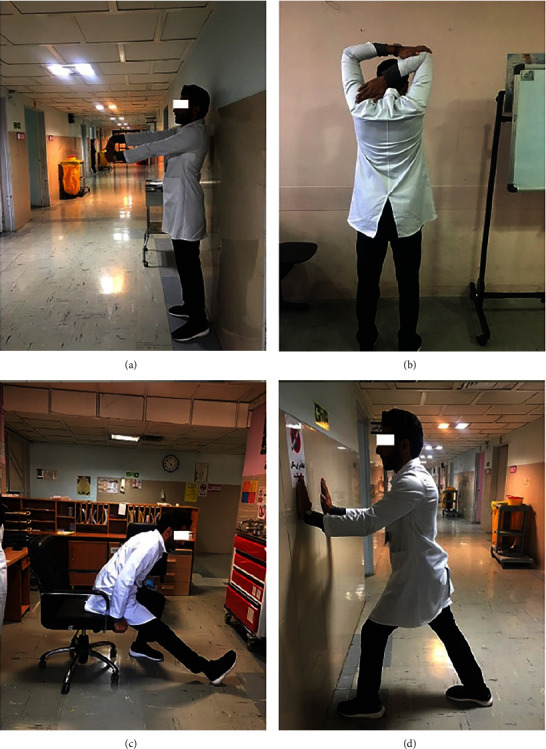
Some stretches used by the study nurses in the workplace: (a) wrist extensor stretch, (b) overhead triceps stretch, (c) sitting hamstring stretch, and (d) standing gastrocnemius-upper-calf stretch [22].

Figure 3 depicts some stretches used by the study nurses in the workplace.

## 4. Discussion

Because the previous study results showed that the prevalence of WMSs and the amount of fatigue were high among hospital nurses, we were encouraged to take an intervention to relieve work-related musculoskeletal complications and fatigue in this working group.

This study aimed to design and use an application to relieve work-related musculoskeletal complications and fatigue among Iranian hospital nurses. The means ± standard deviations of the subjects' age and job tenure were 33.32 ± 6.42 years and 9.70 ± 6.70 years, respectively.

The discussion is presented in two sections as follows.

### 4.1. Stage I: Designing an Application to Present Proper Stretches

After developing the final version of NSB, we tested the usability of the application. User-centered design considers end users' needs and limitations in each stage of the design process by using methods [24]. Examining the usability testing of the NSB from the experts' and study nurses' point of view via the P-USE revealed that the NSB was a useful, easy to use, easy to learn, and satisfactory application to relieve work-related musculoskeletal complications and fatigue among hospital nurses. The ratio of the mean score to the total score in each subscale was equal to or greater than 92.39%. In the development of the USE questionnaire, Lund states that usability consists of usefulness and ease of use for many applications, and usefulness and ease of use are correlated. Each factor, in turn, leads to the user satisfaction and frequency of use. Users appear to have a good sense of what is useable and what is not and can apply their internal metrics across the domains [20]. Studies showed that both end users (nurses and physicians) and IT experts confirmed that the Decision Support System (DSS) was beneficial for increasing the effectiveness of the software. Decision support tools can alert the users about potential problems that might otherwise go unnoticed [24].

### 4.2. Stage II: Using the NSB to Relieve Work-Related Musculoskeletal Complications and Fatigue among Nurses

The findings of the study showed that the highest prevalence of WMSs in the last 7 days among the study nurses was related to the ankles/feet (80.3%), lower back (77.5%), and knees (59.2%), respectively. The prevalence of WMSs in different body regions of the study population was almost similar to that reported among other Iranian working groups, including hospital nurses [9], workers in orthotic and prosthetic workshops [25], healthcare providers [4], hospital attendants [26], office workers [27], and agricultural workers [28].

Based on observation and interviews with hospital nurses, the high prevalence of WMSs among the study nurses can be attributed to the predisposing risk factors, including awkward/static working postures for prolonged periods during patient transfer, strenuous physical demands of the nursing profession, and their poor health and fitness conditioning status.

Our findings demonstrated a significant reduction in the prevalence of WMSs in the last 7 days in different body regions, except for the elbows and knees after the intervention as compared to before it. In addition, it was revealed that the intensity of work-related musculoskeletal pain/discomfort in all body regions, except the knees, was significant before and after the intervention. Although work-related musculoskeletal complications in the elbows and knees decreased after the intervention compared to before, this reduction was not statistically significant.

The comparison of the study groups in the total fatigue and its subscales before and after the intervention showed that the mean score of the total fatigue and its subscales, except “degree of interference with activities of daily living,” were significant before and after the intervention. These findings indicate that using NSB to provide appropriate stretch could effectively relieve musculoskeletal complications in almost all body regions and reduce fatigue among hospital nurses.

Abdelall et al. in their study showed that the web-application microbreak stretches helped to reduce physical pain, discomfort/pain, and fatigue and improve mental focus with minimal disruption among the surgeons [29]. In addition, Tavakkol et al., in their study on the operating room personnel, revealed that regular exercise and physical activity, use of stretching, and gentle exercise could be practical approaches to relieving the WMSs [30].

Zayed et al., in their study, stated that warming up and stretching before performing their nursing duties were a coping strategy for reducing WMSs among the nursing staff [31]. In a study conducted by Hale, it was concluded that stretching throughout the workday could be helpful in reducing WMSs among the workers [32]. In a review study, Gasibat et al. found that stretching improved the joints ROM, which may help people assume more effective work postures and perform tasks more safely, with the best possible body mechanics [33]. Stretching may also help immediately reduce the intensity of pain and stiffness in the muscles and joints [22].

### 4.3. Limitations of the Study

In the current study, there was no control group. Given the data collection by self-report, the findings should be interpreted cautiously. Moreover, this study was performed among governmental hospital nurses in Shiraz. Therefore, the results might not be generalizable to other working groups.

## 5. Conclusion

The findings of the current study demonstrated that NSB could be an effective and practical approach to relieve work-related musculoskeletal complications and fatigue among hospital nurses. In addition, the usability testing of the NSB showed that it was a useful, easy to use, easy to learn, and satisfactory application.

### 5.1. Recommendations

Finally, we recommend that the newly designed application (NSB) should be used to reduce musculoskeletal pain and fatigue among hospital nurses. A practical recommendation for nurses is to install NSB on their smartphones and perform various stretches for 5 to 10 minutes every hour. They are also advised to hold stretches for 30 seconds at an intensity sufficient to elicit a feeling of strong stretch, not pain.

## Figures and Tables

**Table 1:** Usability testing of the NSB from the experts' point of view using the P-USE questionnaire (*n* = 9).

Subscale	Mean ± SD	Percentage of the total score
Usefulness	55.16 ± 4.89	98.50
Ease of use	75.66 ± 2.32	98.25
Ease of learning	27.66 ± 0.91	98.78
Satisfaction	49.00 ± 1.34	100.00
Total	207.5 ± 7.11	98.80

**Table 2:** Some personal/occupational details of the participants (*n* = 71).

Quantitative variables	Mean ± SD	Min–max
Age (years)	33.32 ± 6.42	24–50
Height (cm)	164.56 ± 7.07	153–184
Weight (kg)	65.28 ± 10.00	45–94
Body mass index (kg·m^−2^)	24.10 ± 3.36	17.30–34.48
Job tenure (years)	9.70 ± 6.70	1–28
Working h/day	8.19 ± 1.51	6.67–13.00

Qualitative variables	No. (%)	
Gender		
Male	15 (21.1)	
Female	56 (78.9)	
Marital status		
Single	25 (35.2)	
Married	46 (64.8)	
Smoking		
Yes	1 (1.4)	
No	70 (98.6)	
Shift work		
Yes	61 (85.9)	
No	10 (14.1)	
Type of employment		
Formal^∗^	36 (50.7)	
Contractor^†^	35 (49.3)	

^∗^Permanent employment. ^†^Transient employment based on a contract.

**Table 3:** Comparison of the study groups regarding the prevalence of WMSs in the last 7 days in different body regions before and after the intervention (*n* = 71).

Body region	Prevalence of WMSs in the last 7 days		*p* value^∗^
	Before intervention no. (%)	After intervention no. (%)	
Neck	36 (50.7)	23 (32.4)	0.002
Shoulders	39 (54.9)	17 (23.9)	<0.001
Elbows	5 (7.0)	4 (5.6)	0.419
Wrists/hands	29 (40.8)	6 (8.5)	<0.001
Upper back	40 (56.3)	15 (21.1)	<0.001
Lower back	55 (77.5)	20 (28.2)	<0.001
Thighs	18 (25.4)	0 (0.00)	<0.001
Knees	42 (59.2)	38 (53.52)	0.383
Ankles/feet	57 (80.3)	20 (28.2)	<0.001
WMSs at least in one body region	71 (100)	65 (91.5)	0.031

^∗^McNemar's test.

**Table 4:** Comparison of the study groups regarding the intensity of work-related musculoskeletal pain/discomfort in different body regions before and after the intervention (*n* = 71).

Body region	The intensity of work-related musculoskeletal pain/discomfort		*p* value^∗^
	Before intervention *Mdn* (IQR)^†^	After intervention *Mdn* (IQR)^†^	
Neck	2 (0.5–3.5)	1 (0.5–2.0)	<0.001
Shoulders	1.5 (0.0–3.5)	1 (0.0–1.5)	<0.001
Elbows	0 (0.0–1.5)	0 (0.0–0.5)	0.002
Wrists/hands	2 (0.5–3.5)	1 (0.5–3.5)	<0.001
Upper back	2 (0.5–4.0)	1 (0.0–2.0)	<0.001
Lower back	2.5 (1.0–4.5)	1.5 (1.0–2.0)	<0.001
Thighs	0.5 (0.0–2.0)	0 (0.0–0.5)	<0.001
Knees	2.5 (1.0–4.0)	2 (0.5–2.0)	0.383
Ankles/feet	2.5 (1.5–5.0)	1.5 (0.5–2.5)	<0.001
WMSs at least in one body region	1.94 (1.22–2.66)	0.83 (0.61–1.38)	0.029

^∗^Wilcoxon signed-rank. ^†^Median (interquartile range).

**Table 5:** Comparison of the study groups regarding the total fatigue and its subscales before and after the intervention (*n* = 71).

P-MAF subscale	Before intervention M ± SD^†^	After intervention M ± SD^†^	Range	*p* value^∗^
Degree and severity	7.60 ± 1.57	5.84 ± 1.24	1–10	<0.001
Distress that it causes	7.37 ± 1.93	5.77 ± 1.40	1–10	<0.001
Degree of interference with activities of daily living	1.53 ± 0.34	1.50 ± 0.26	1–10	0.630
Timing of fatigue	4.40 ± 1.76	5.42 ± 1.26	1–10	<0.001
Total fatigue/global fatigue index	27.31 ± 4.52	23.05 ± 3.35	1–50	<0.001

^∗^Paired sample *t*-test. ^†^Mean ± standard deviation.

**Table 6:** Usability testing of the NSB from the nurses' point of view via the P-USE questionnaire.

Subscale	Mean ± SD	Min–max	Percentage of the total score
Usefulness	51.74 ± 4.74	8–56	92.39
Ease of use	76.14 ± 1.45	11–77	99.88
Ease of learning	27.81 ± 0.54	4–28	99.32
Satisfaction	48.54 ± 1.13	7–49	99.06
Total	204.25 ± 6.24	30–210	97.26

## Data Availability

The data used to support the findings of this study are available from the corresponding author upon request.
